# Influence of Annealing on the Dielectric Properties of Zn-SiO_2_/Si Nanocomposites Obtained in “Hot” Implantation Conditions

**DOI:** 10.3390/nano12193449

**Published:** 2022-10-02

**Authors:** Tomasz N. Kołtunowicz, Karolina Czarnacka, Piotr Gałaszkiewicz, Fadei F. Komarov, Maxim A. Makhavikou, Oleg V. Milchanin

**Affiliations:** 1 Department of Electrical Devices and High Voltage Technology, Lublin University of Technology, 20-618 Lublin, Poland; 2 Department of Technology Fundamentals, University of Life Sciences in Lublin, 20-950 Lublin, Poland; 3 Institute of Applied Physics Problems, Belarusian State University, 220030 Minsk, Belarus

**Keywords:** nanocomposite, ion implantation, impedance spectroscopy, charge transport

## Abstract

This paper presents the results of AC electrical measurements of Zn-SiO_2_/Si nanocomposites obtained by ion implantation. Implantation of Zn ions was carried out into thermally oxidized p-type silicon substrates with energy of 150 keV and fluence of 7.5 × 10^16^ ion·cm^−2^ at a temperature of 773 K, and is thus called implantation in “hot” conditions. The samples were annealed in ambient air for 60 min at 973 K. Electrical measurements of Zn-SiO_2_/Si nanocomposites were carried out before and after annealing. Measurements were performed in the temperature range from 20 K to 375 K. The measurement parameters were the resistance *R*_p_, the capacitance *C*_p_, the phase shift angle θ and the tangent of loss angle tanδ, as a function of the frequency in the range from 50 Hz to 5 MHz. Based on the characteristics σ(*f*) and the Jonscher power law before and after sample annealing, the values of the exponent *s* were calculated depending on the measurement temperature. Based on this, the conductivity models were matched. Additionally, the real and imaginary parts of the dielectric permittivity were determined, and on their basis, the polarization mechanisms in the tested material were also determined.

## 1. Introduction

Ion implantation is one of the most flexible methods of producing nanocomposites with metallic and semiconductor nanoparticles in the SiO_2_ matrix due to high purity of the process, precise control of the implanted ions’ fluence, the spatial distribution of implanted atoms, etc. [1,2,3,4]. However, the main advantage of this method is its full compatibility with modern silicon microelectronics. At high implantation fluences, the concentration of incorporated impurities in the matrix significantly exceeds the limit of equilibrium solubility, and the formation of nanoparticles occurs.

Compared to other techniques, such as chemical vapor deposition (CVD) [5], molecular beam epitaxy (MBE) [6], sol–gel synthesis [7] and pulsed laser deposition [8], ion implantation has several important advantages, including no synthesis-related impurities and precise control of the introduced metal ions and their depth distribution in the dielectric matrix [9]. Accordingly, nanoparticles of metals and metal oxides, such as copper (Cu) [10], gold (Au), nickel (Ni) [11], silver (Ag) [12], cobalt (Co) [13], etc., were synthesized by ion implantation in dielectrics such as SiO_2_ [14], Al_2_O_3_ [15], and even in MgO [16,17] or CeO_2_ [13]. According to the literature sources, in the SiO_2_ matrix, the implanted ions form spherical nanoparticles with a random distribution [14]. In particular, zinc (Zn) and zinc oxide (ZnO) nanoparticles produced in SiO_2_ by ion implantation are popular due to their present and possible applications in high-performance optoelectronics [18,19,20].

The dielectric properties of nanomaterials are important characteristics for their potential applications in capacitors, sensors and memory devices. The influence of frequency on the dielectric behavior and AC conductivity of nanomaterials provides key information on the conductive phenomena in these materials [21,22]. It should be noted that the dielectric properties and electric transport of nanomaterials are different from the properties of materials on a micro- or macrometric scale, mainly due to an increase in the number of atoms or ions between phases, and also due to the fact that many structural defects are introduced in or near the grain boundaries. AC measurements of nanocomposites’ electrical properties have been extensively conducted for granular metal–dielectric nanocomposites, with SiO_2_ [23], Al_2_O_3_ [24,25], CaF_2_ [26,27] and PZT [27,28,29] dielectrics used as matrix materials, and CoFeZr ferromagnetic alloy [24,25,26,27,28,29] and copper [23,30] used as nanofiller.

In this work, impedance spectroscopy was used to characterize and compare the electrical properties of a nanocomposite with the silicon oxide matrix in which metallic zinc nanograins (nanoclusters) were formed by ion implantation. As a result, it gives an opportunity to determine mechanisms of electric charge transfer in this material and to study other phenomena conditioned by these mechanisms.

## 2. Experimental

The initial SiO_2_/p-type Si samples 2 cm × 2 cm in size with an electrical resistivity of 10 Ω·cm for a silicon wafer were cut from thermally oxidized Si substrates. The thickness of the SiO_2_ layer, as measured by transmission electron microscopy in cross-section geometry by an analytical electron microscope Hitachi H-800 operating at 200 keV, was about 600 nm. These samples were implanted at the temperature of 773 K with 150 keV-Zn ions to the fluence of 7.5 × 10^16^ ion·cm^−2^. Therefore, this regime is called implantation in “hot” conditions. Afterwards, the samples were annealed at 973 K for 60 min in ambient air. Zn concentration depth distributions of the implanted and annealed samples were analyzed by Rutherford backscattering spectrometry (RBS) using 1.4 MeV He ions with a registration angle of 170 degrees. Information on depth distribution of Zn atoms’ concentration was obtained by processing the RBS spectra using the SIMNRA software package [31].

The measured samples consisted of the p-type silicon substrate on which the 600 nm SiO_2_ layers were formed by oxidation in dry ambient O_2_. After this process, the Zn^+^ ion implantation was performed and a part of the samples was annealed.

A thin layer of silver paste was applied to the tested nanocomposite samples in order to avoid the negative impact of point contact during the measurements. Electrical measurements of Zn-SiO_2_/Si nanocomposites were carried out through the sample (in a capacitor system), so the contacts were placed on both sides of the sample, leaving a distance of approx. 1 mm from its edge.

**Figure 1:**
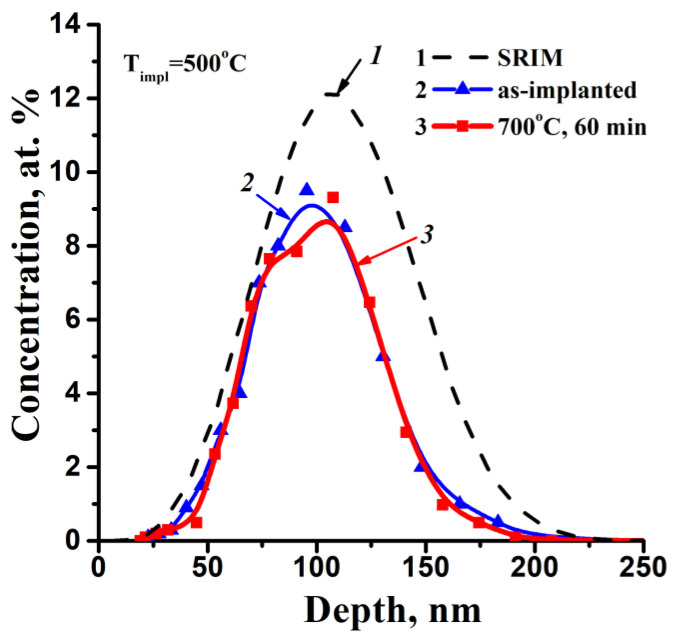
Simulated (SRIM-2013) (curve 1) and calculated from RBS spectra depth profiles of impurity concentration in SiO_2_ after Zn^+^ implantation (150 keV, 7.5 × 10^16^ cm^−2^) (curve 2) and subsequent annealing at 973 K (700 °C) for 60 min (curve 3).

It should also be mentioned that a part of implanted atoms can penetrate the SiO_2_/Si interface due to radiation-enhanced diffusion in the process of ion implantation and sample annealing. However, a concentration of such Zn atoms in the tail part of depth distributions in Figure 1 is under the mentioned resolution of the RBS method (about 5 × 10^18^ at/cm^3^). Such a level of silicon dioxide doping can, to a significant degree, change the conductivity properties of this deep part of implanted oxide, as well.

Liquid helium cooling was applied using a cryostat. This made it possible to conduct measurements with temperature regulation with an accuracy of 0.002 K. Additionally, a vacuum chamber was used, in which the samples were mounted. It helped stabilize the temperature and prevent the samples from damping. Thus, the measurements were carried out in a vacuum at the level of 0.2 atm. The temperature in the chamber was cooled in a closed circuit using a helium compressor. The temperature control in the chamber was ensured by a silicon sensor, a LakeShore 335 temperature controller, and a heater located in the cryostat head connected to the controller. Measurements were performed in the temperature range from 20 K to 375 K, with the step of 2 K in the range of 20–40 K, with the step of 3 K in the range of 40–151 K and with the step of 7 K in the range of 151–375 K.

Measurements of electrical parameters were carried out using HIOKI impedance meters. The measurement parameters were the resistance *R*_p_, capacitance *C*_p_, the phase shift angle θ and the tangent of the loss angle tanδ, as a function of the frequency in the range of 50 Hz to 5 MHz. The test stand is controlled by a computer to which the meters and a temperature controller are connected. The measured results are saved on the computer′s hard disk in xls format [32].

## 3. Results and Discussion

Figure 1 shows the simulated (SRIM’13 [33]) depth distribution profile of implanted Zn atoms and the profiles calculated from experimental RBS spectra. Computer simulation gave a Gaussian-type Zn concentration depth profile with a peak concentration of about 12 at % at the depth of ~113 nm in the oxide matrix (Figure 1, curve 1). “Hot” implantation into SiO_2_ (Figure 1, curve 2) leads to a 1.3-fold reduction in the maximum impurity concentration in comparison with the computer simulation data. The impurity loss during ion implantation amounts to 38%. Annealing (973 K, 60 min) results in an insignificant diffusion of implanted impurity to the sample surface (Figure 1, curve 3).

**Figure 2:**
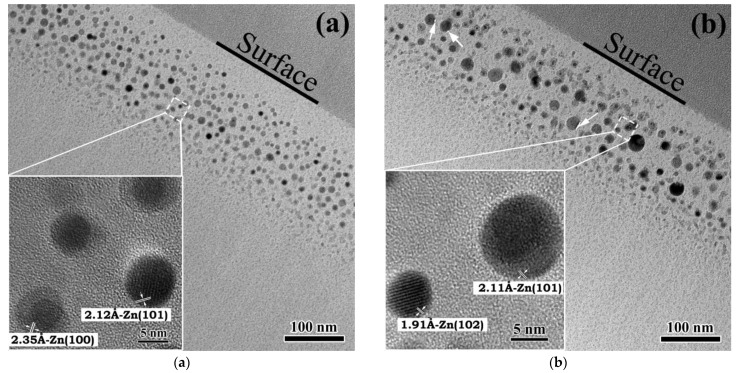
XTEM images of SiO_2_ (600 nm)/Si samples implanted with Zn^+^ (150 keV, 7.5 × 10^16^ ions/cm^2^): (**a**) as-implanted sample, and the samples after annealing at 973 K for (**b**) 60 min. The insets depict the HRTEM nanoparticle images.

Figure 2 shows the XTEM and HRTEM images (insets) of Zn nanoparticles embedded in the SiO_2_ matrix. “Hot” implantation leads to the formation of small clusters just after zinc implantation (see Figure 2a). The absence of any nanoclusters in the subsurface region (up to 40 nm) of the SiO_2_ layers proves the minor zinc atoms’ diffusion to the surface at elevated temperatures up to 973 K. Underneath the cluster-free region, the layer with small clusters (2–15 nm) is located in the depth range of 40–200 nm.

The bigger clusters 10–15 nm in size are concentrated at the depth of 100–110 nm (nearly *R*_p_; *R*_p_ is the projected range of implanted atoms). The crystalline nature of the precipitates is proved by the presence of Moiré contrast in the HRTEM images (see Figure 2a,b, insets). The interplanar spacing 2.12 Å measured for dark contrast precipitate (Figure 2a, inset) is in agreement with the tabulated value of 2.09 Å for the (101) planes of the hexagonal Zn. Heat treatment at 973 K for 60 min (Figure 2b) results in a substantial structural transformation of the implanted layer. One can see two spatial separated layers. The small Zn nanoparticles surrounded by the oxide shells (see Figure 2b, inset) at the depth range of 30–100 nm should be noted. The calculated value of the interplanar spacing of the oxide shells is 1.91 Å. It corresponds to the distance between (102) planes of the hexagonal ZnO phase. In addition, the formation of voids is observed at the “Zn nanocluster–SiO_2_ matrix” interface (Figure 2b, shown by arrows).

**Figure 3:**
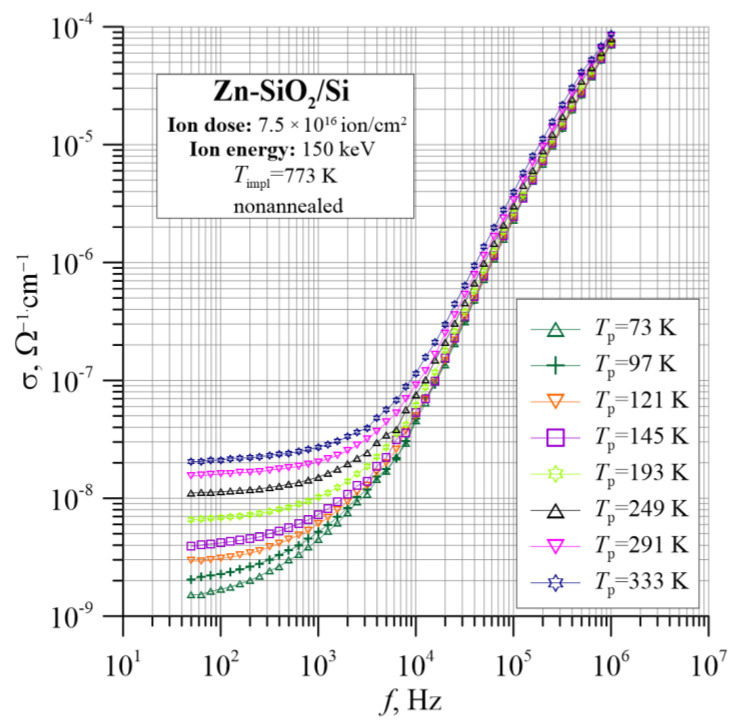
Temperature–frequency dependence of the conductivity σ(*f*, *T*_p_) for the Zn-SiO_2_/Si nanocomposite immediately after preparation under “hot” implantation conditions (150 keV, 7.5 × 10^16^ ion/cm^2^, *T*_impl_ = 773 K).

Figure 3 shows the temperature–frequency dependence of the Zn-SiO_2_/Si nanocomposite conductivity immediately after implantation at the temperature *T*_impl_ = 773 K with 150 keV-Zn ions to the fluence of 7.5 × 10^16^ ion/cm^2^. In the range of lower frequencies, a weak conductivity dependence on frequency can be seen, corresponding to the DC conductivity σ_dc_. In this frequency range, a strong temperature dependence characteristic is observed for dielectric conductivity. At the same time, as the temperature rises, the plateau of DC conductivity extends to higher frequencies. With a further increase in frequency, the conductivity increases significantly (almost 5–6 orders of magnitude), but shows a poor temperature dependence. Such a conductivity behavior with frequency may indicate a hopping mechanism of charge transfer in the tested material [34,35,36]. The increase in AC conductivity with frequency is due to the relaxation of moving electrons. As the frequency increases, the charge carriers are forced to jump between the located states, which causes a decrease in activation energy.

**Figure 4:**
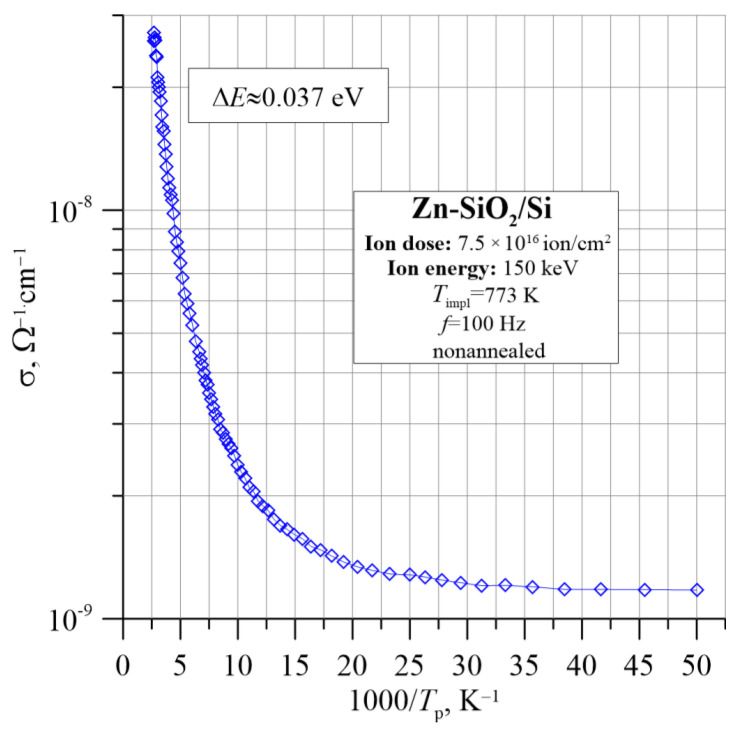
The Arrhenius dependence of the conductivity σ for the Zn-SiO_2_/Si nanocomposite immediately after preparation under “hot” implantation conditions (150 keV, 7.5 × 10^16^ ion/cm^2^, *T*_impl_ = 773 K).

Figure 4 shows the Arrhenius dependence for conductivity determined at the frequency *f* = 100 Hz. The diagram presents three ranges of change in conductivity with temperature, which may indicate at least two ways of the charge transporting in the tested material. In the range of low temperatures, to approx. 35 K, the conductivity is practically constant and does not show an activating character. It is related to the carrier hopping near the Fermi level *E*_F_. In the range from 35 K to about 135 K, conductivity changes are non-linear and it is difficult to determine the electron activation energy on the basis of the Arrhenius law. In the range of high temperatures, a linear change in conductivity with temperature is visible, which fulfills the Arrhenius law, making it possible to calculate the activation energy of the conductivity. Its value is approximately Δ*E*_1_ ≈ 0.037 eV. This confirms the presence of a thermally activated hopping conductivity [22,23].

**Figure 5:**
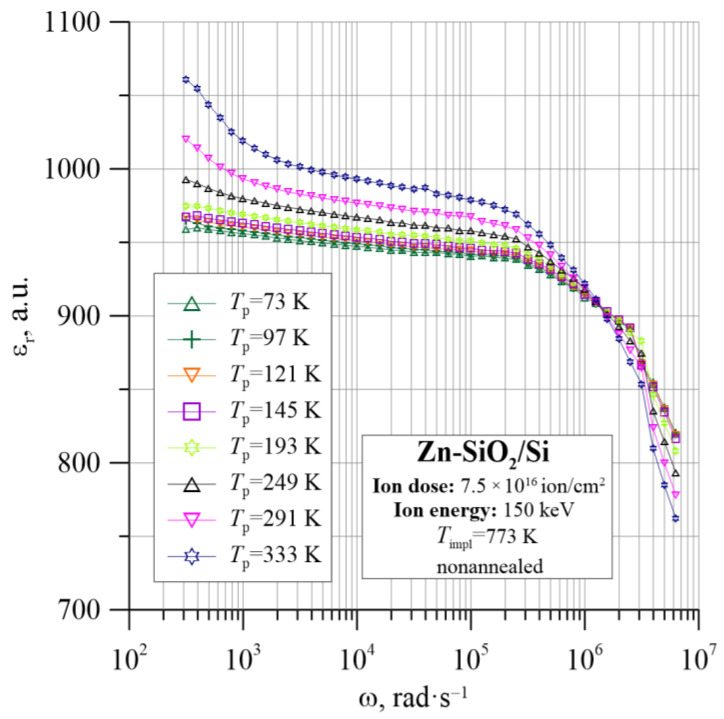
Temperature dependence of the real part of dielectric permittivity ε_r_ for the Zn-SiO_2_/Si nanocomposite immediately after preparation under “hot” implantation conditions (150 keV, 7.5 × 10^16^ ion/cm^2^, *T*_impl_ = 773 K).

**Figure 6:**
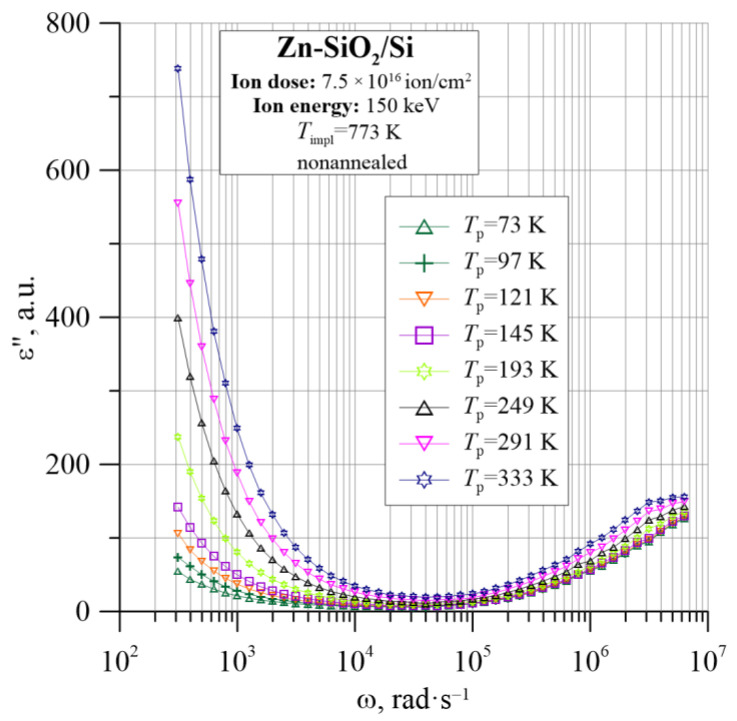
Temperature dependence of the imaginary part of dielectric permittivity ε″ for the Zn-SiO_2_/Si nanocomposite immediately after preparation under “hot” implantation conditions (150 keV, 7.5 × 10^16^ ion/cm^2^, *T*_impl_ = 773 K).

**Figure 7:**
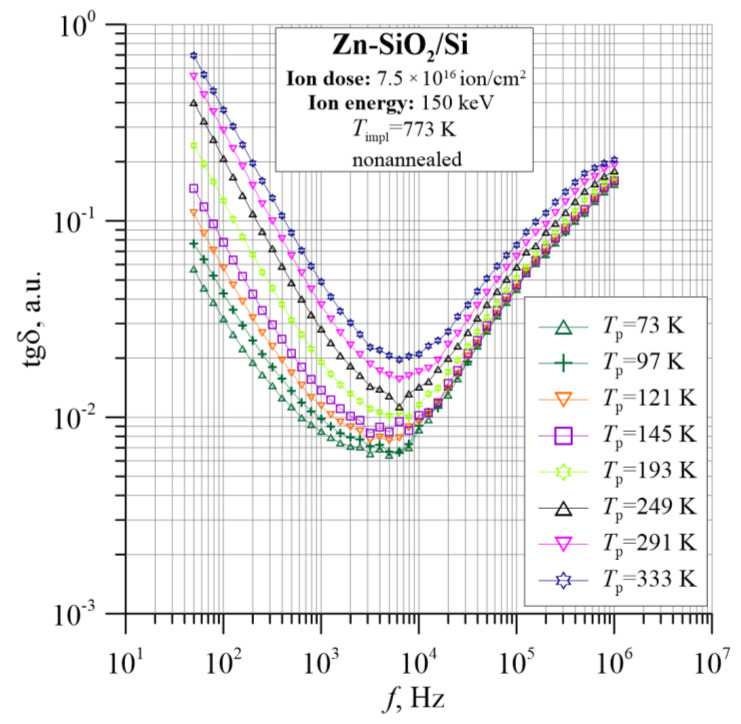
Temperature dependence of the tangent of dielectric loss angle tgδ for the Zn-SiO_2_/Si nanocomposite immediately after preparation under “hot” implantation conditions (150 keV, 7.5 × 10^16^ ion/cm^2^, *T*_impl_ = 773 K).

Figure 5, Figure 6 and Figure 7 show the dielectric properties of the tested Zn-SiO_2_/Si nanocomposite, which were measured in the frequency range from 50 Hz to 5 MHz at temperatures from 20 K to 375 K. The real dielectric component ε_r_ shows a sharp decrease with frequency in the low and high frequencies. In the range of intermediate frequencies, ε_r_ decreases slightly or is practically constant. Accordingly, the imaginary part of the dielectric permittivity ε″ in the low- and intermediate-frequency ranges rapidly decreases with increasing frequency, reaching a Debye-like relaxation peak at angular frequencies of about 4 × 10^6^ rad/s. The low-frequency section of the ε″ relationship is related to the DC conductivity. The tangent of the dielectric loss angle tanδ is high, with a temperature-dependent minimum ranging from 6 × 10^−3^ to 2 × 10^−2^ at a frequency of about 10^4^ Hz. ε_r_ and ε″ frequency dependencies are used later in this work to determine the Cole–Cole relationship.

**Figure 8:**
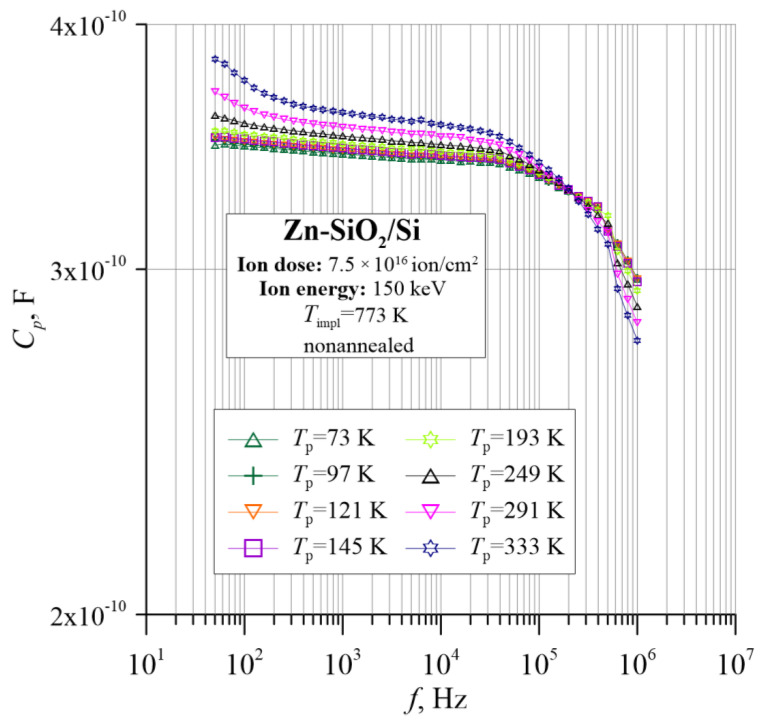
Temperature dependence of the capacitance *C*_p_ for the Zn-SiO_2_/Si nanocomposite immediately after preparation under “hot” implantation conditions (150 keV, 7.5 × 10^16^ ion/cm^2^, *T*_impl_ = 773 K).

**Figure 9:**
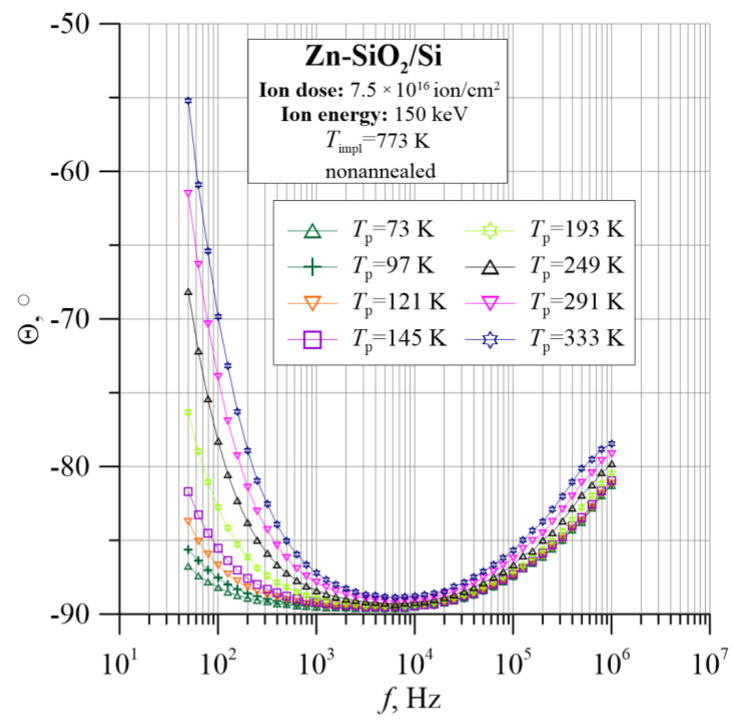
Temperature dependence of the phase shift angle θ for the Zn-SiO_2_/Si nanocomposite immediately after preparation under “hot” implantation conditions (150 keV, 7.5 × 10^16^ ion/cm^2^, *T*_impl_ = 773 K).

Figure 8 and Figure 9 show the frequency–temperature dependences of the capacitance *C*_p_ and the phase shift angle θ. On their basis, it can be concluded that the material has a capacitive character because the *C*_p_ dependence is practically independent of frequency and temperature. Moreover, the phase shift angle in the entire measuring range is negative. Additionally, the angle θ in the range of low frequencies decreases, reaching a value close to −90°, which is characteristic of the parallel RC circuit.

**Figure 10:**
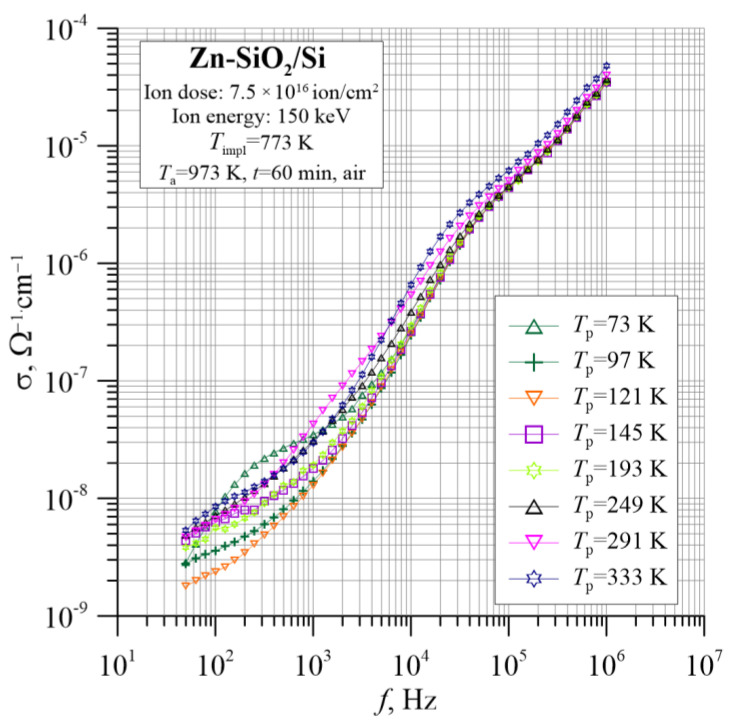
Temperature–frequency dependence of the conductivity σ(*f*, *T*_p_) for the Zn-SiO_2_/Si nanocomposite prepared under “hot” implantation conditions (150 keV, 7.5 × 10^16^ ion/cm^2^, *T*_impl_ = 773 K) after annealing.

**Figure 11:**
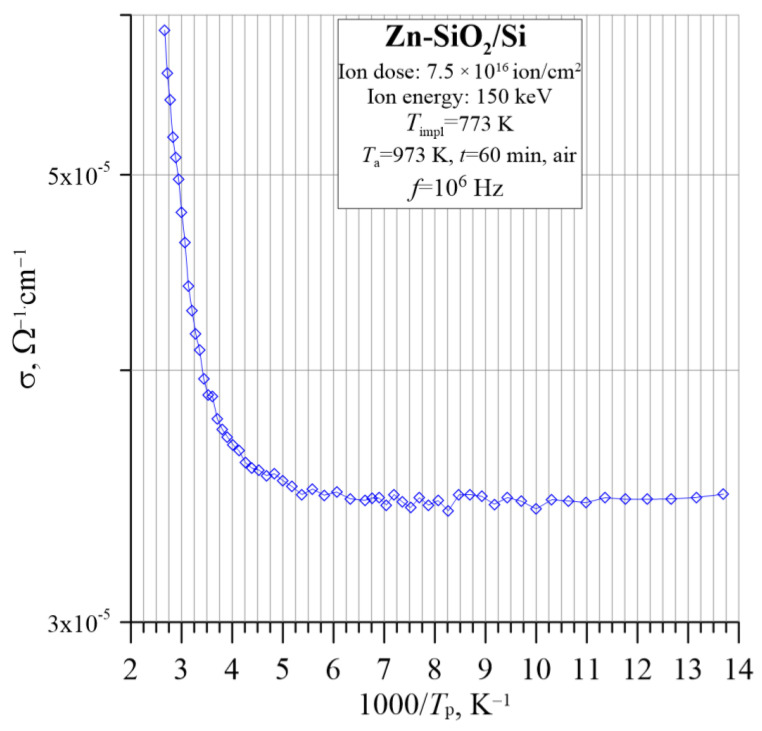
The Arrhenius dependence of the conductivity σ for the Zn-SiO_2_/Si nanocomposite prepared under “hot” implantation conditions (150 keV, 7.5 × 10^16^ ion/cm^2^, *T*_impl_ = 773 K) after annealing for frequency *f* = 10^6^ Hz.

The situation changes significantly after annealing. Figure 10 shows a graph of the temperature–frequency dependence of the conductivity for the Zn-SiO_2_/Si sample obtained in the “hot” implantation conditions subjected to annealing at 974 K in ambient air for 60 min. The increase in conductivity with a frequency of up to 6 orders of magnitude is clearly visible. However, in the low- and medium-frequency range, the conductivity does not show an orderly dependence on temperature. This can be seen in the Arrhenius graphs of conductivity shown in Figure 11. Only in the high-frequency range, the conductivity increases with temperature, showing dielectric conduction behavior.

At high frequencies (Figure 11), three ranges of conductivity change with temperature can be distinguished. The first one is up to approx. 200 K, where the conductivity does not depend on the measurement temperature and is related to the hopping of carriers near the Fermi level *E*_F_. The second one is at temperatures up to approx. 280 K, where changes in conductivity are non-linear. The third region of high temperatures is above 280 K, where there is a linear change according to the Arrhenius law. In this temperature range, it was possible to determine the thermal activation energy of electrons, and it amounts to Δ*E*_2_ ≈ 0.060 eV.

**Figure 12:**
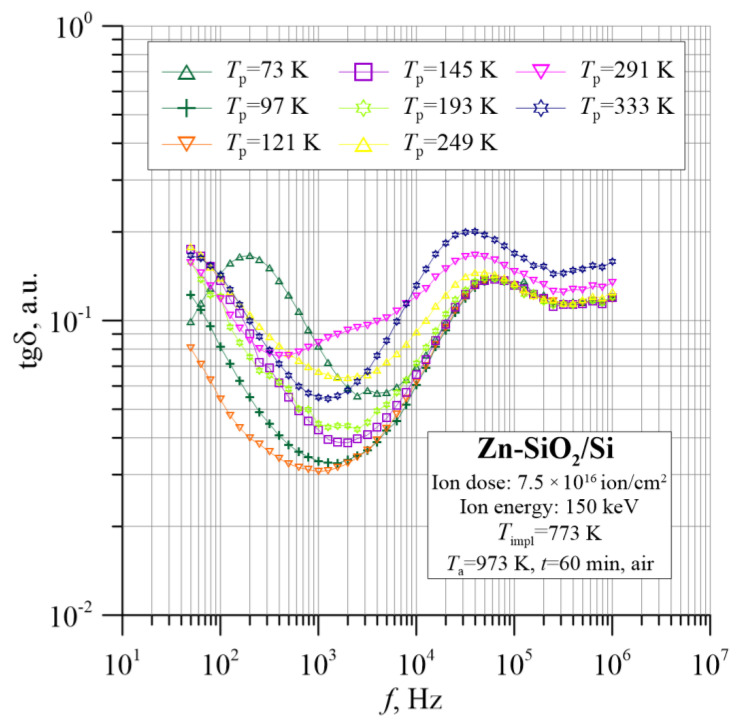
Temperature–frequency dependence of the tangent of dielectric loss angle tgδ(*f*, *T*_p_) for the Zn-SiO_2_/Si nanocomposite prepared under “hot” implantation conditions (150 keV, 7.5 × 10^16^ ion/cm^2^, *T*_impl_ = 773 K) after annealing.

**Figure 13:**
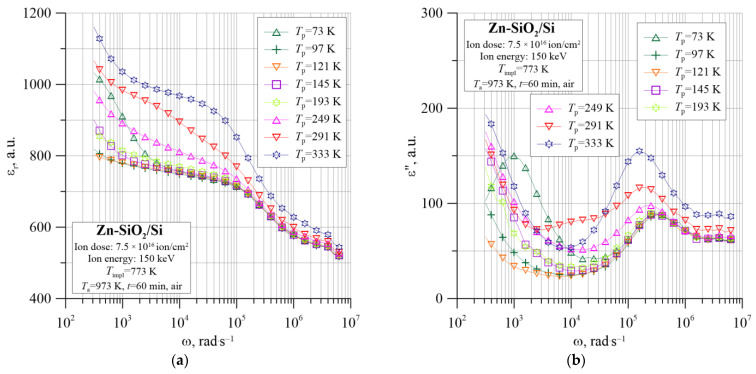
Temperature–frequency dependence of the real (**a**) and imaginary (**b**) part of dielectric permittivity for the Zn-SiO_2_/Si nanocomposite prepared under “hot” implantation conditions (150 keV, 7.5 × 10^16^ ion/cm^2^, *T*_impl_ = 773 K) after annealing.

**Figure 14:**
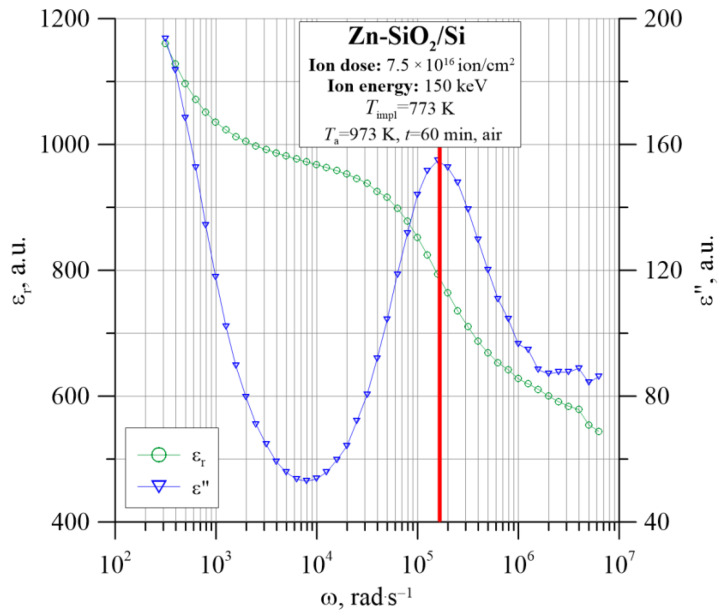
Comparison of the real and imaginary dielectric permittivity as a function of frequency measured at *T*_p_ = 333 K.

**Figure 15:**
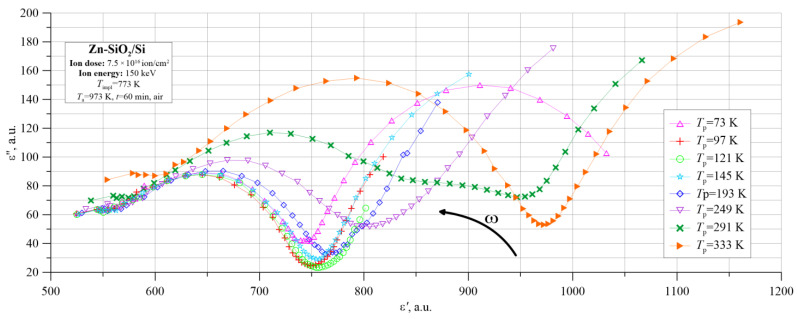
Cole–Cole diagram of Zn-SiO_2_/Si nanocomposite obtained in the conditions of “hot” implantation (150 keV, 7.5 × 10^16^ ion/cm^2^, T_impl_ = 773 K) and annealed at the temperature of *T*_a_ = 973 K.

**Figure 16:**
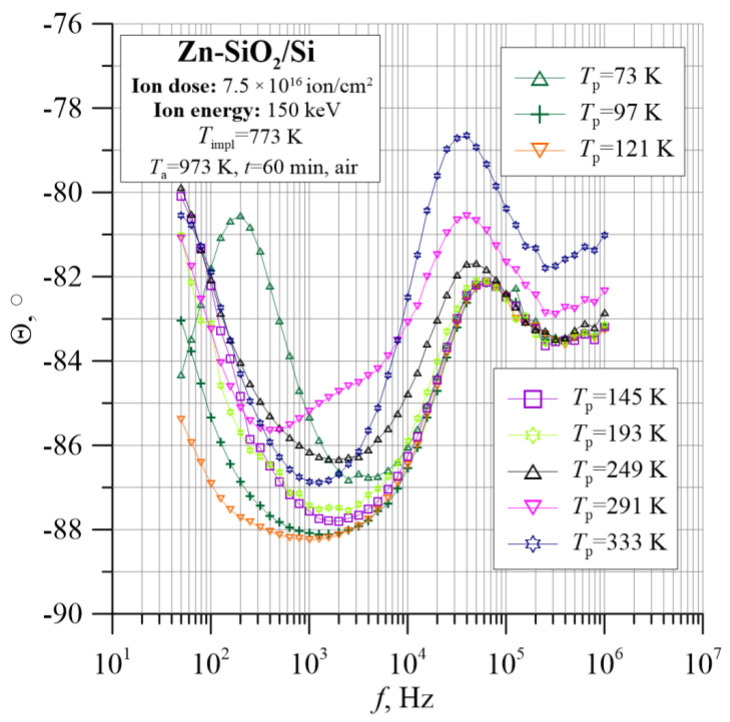
Temperature–frequency dependence of phase shift angle θ(*f*, *T*_p_) for the Zn-SiO_2_/Si nanocomposite prepared under “hot” implantation conditions (150 keV, 7.5 × 10^16^ ion/cm^2^, *T*_impl_ = 773 K) after annealing.

This theory is confirmed by the temperature–frequency dependencies of the dielectric parameters shown in Figure 12, Figure 13, Figure 14, Figure 15 and Figure 16. The tangent of the dielectric loss angle tanδ (Figure 12) changes parabolically with the frequency, which indicates a relaxation loss mechanism.

Figure 13 shows the frequency–temperature dependence of the real ε_r_ (Figure 13a) and the imaginary ε″ (Figure 13b) components of dielectric permittivity. The analysis of these graphs proves the hopping charge transfer between the closest neighbors, creating electric dipoles.

First, there is a characteristic maximum in the graph ε″(*f*) in the frequency range above 10^5^ Hz. Second, the frequency at which the peak occurs has the same value as the inflection frequency of the descending curve ε_r_(*f*), as shown in Figure 13a. Additionally, in the range of low frequencies, the permeability ε″ shows another polarization mechanism, which is especially visible for the lowest temperatures. A second relaxation peak appears which, at higher temperatures, is likely to occur at lower frequencies than the measuring range. In the case of the annealed sample, the decrease in ε_r_ with frequency is not related to the DC conductivity (as in the case of a nonannealed sample), because the characteristic σ(*f*) shows a strong frequency dependence also in the lowest range. It is more likely that there is a space charge polarization according to the Maxwell–Wagner model at the metal–dielectric interface for larger metallic nanoparticles and at the dielectric–silicon substrate interface. Relaxation according to this mechanism requires higher activation energies and a longer time (i.e., low frequency). A comparison of the real and imaginary dielectric permeability as a function of frequency measured at *T*_p_ = 333 K is shown in Figure 14.

This phenomenon is clearly visible in the Cole–Cole diagrams presented in Figure 15. For the entire temperature range, characteristic semi-circles corresponding to the electric dipoles’ creation due to hopping charge transfer between the closest neighbors are visible, and in the lower frequency range, fragments of the semi-circle can be seen, confirming the Maxwell–Wagner model occurring at low frequencies. At the lowest temperatures, two characteristic semicircles are visible, corresponding to the peaks in the graph ε″(*f*).

**Figure 17:**
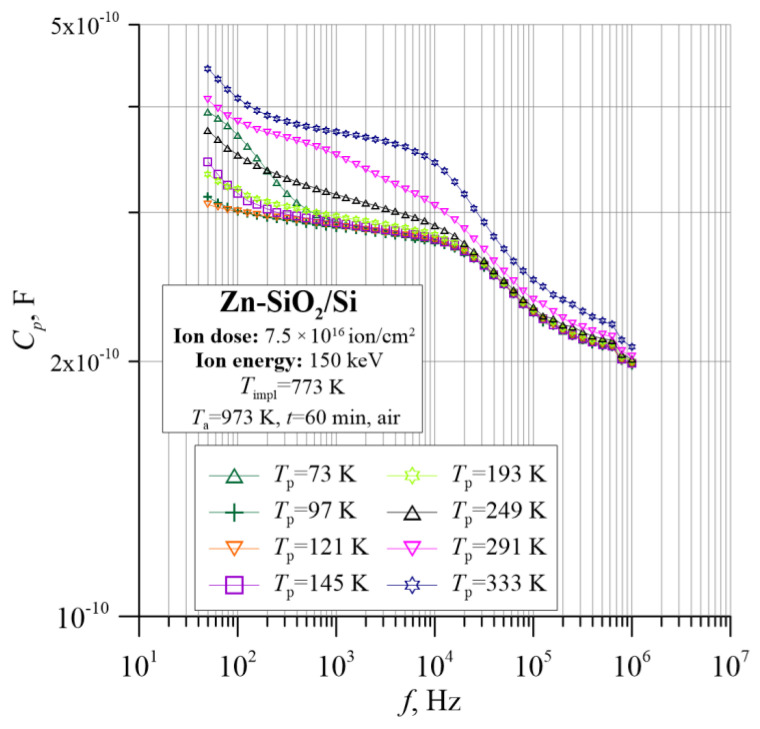
Temperature–frequency dependence of capacity *C*_p_(*f*, *T*_p_) for the Zn-SiO_2_/Si nanocomposite prepared under “hot” implantation conditions (150 keV, 7.5 × 10^16^ ion/cm^2^, *T*_impl_ = 773 K) after annealing.

The analysis of the tested sample is complemented by information about its capacitive behavior, as shown in Figure 16 and Figure 17. In the diagram of the frequency–temperature dependence of the capacitance (Figure 17), the capacitance at the lowest frequencies decreases sharply, and then its value decreases gently, and then decreases again abruptly. This is related to the previously described mechanisms of polarization in the material. Additionally, the phase shift angle presented in Figure 16 shows negative values over the entire measuring range, which proves the capacitive nature of the material.

## 4. Conclusions

A series of Zn-SiO_2_ nanocomposites produced by the implantation of Zn ions into the SiO_2_/Si substrate was selected for analysis, which was carried out with ion energy of 150 keV and fluence of 7.5 × 10^16^ ion·cm^−2^ at a temperature of *T*_impl_ = 773 K. Due to the high temperature of the process, it was described as implantation in “hot” conditions. The samples were annealed in ambient air for 60 min at a temperature of 973 K. The AC measurements were carried out in the temperature range from 20 K to 373 K and in the frequency range from 50 Hz to 1 MHz.

Before annealing, the conductivity increases with increasing frequency by almost 6 orders of magnitude. Based on this, the conductivity model was matched. According to the model, dipoles are formed, which change their orientation under the influence of further electron hops. The analysis of Arrhenius plots of conductivity for the frequency of 100 Hz showed that at temperatures below 35 K, electron hopping takes place near the Fermi level. In the temperature range above 200 K, the conductivity increases linearly with increasing temperature. This is evidence of thermally activated electron hopping.

The annealing of the Zn-SiO_2_/Si nanocomposite sample at the temperature of 973 K caused significant changes in the nature of the σ(*f*) dependence. Three ranges for the change in the slope of the almost rectilinear portions of the σ(*f*) curve are visible.

The nature of the tangent of the dielectric loss angle as a function of the frequency of the nanocomposite sample, both before and after annealing, confirms the occurrence of the relaxation loss mechanisms in the tested material. The analysis of frequency–temperature diagrams of the real ε_r_ and imaginary ε″ component of dielectric permittivity proves the hopping model of charge transfer. It is related to the hopping charge transfer between the closest neighbors’ potential wells and the creation of electric dipoles. In the case of an annealed sample, there is an additional low-frequency space charge polarization at the metal–dielectric interface for larger metallic precipitates and at the dielectric–silicon substrate interface according to the Maxwell–Wagner model. The Zn-SiO_2_/Si nanocomposite obtained in the conditions of “hot” implantation in the entire temperature and frequency range shows a capacitive nature.

## Data Availability

Not applicable.
